# Lung Flare Care: Development of a web resource to improve recovery after COPD exacerbations: A mixed methods study

**DOI:** 10.1371/journal.pone.0324468

**Published:** 2025-05-22

**Authors:** Alethea Kavanagh, Ruben Hopmans, Carla Gordon, Georgia Roberts, Lauren Palmer, Chooi-Ee Lai, Amy Catlin, Rachel Smith, Marielle Collings, Jessica DeGaris, Christian Osadnik

**Affiliations:** 1 Department of Physiotherapy, Monash University, Frankston, Victoria, Australia; 2 Department of Physiotherapy, Monash Health, Melbourne, Victoria, Australia; 3 Monash Lung Sleep Allergy & Immunology, Monash Health, Melbourne, Victoria, Australia; AstraZeneca Pharmaceuticals LP, UNITED STATES OF AMERICA

## Abstract

Recovery from acute exacerbations of chronic obstructive pulmonary disease (AECOPD) is often sub-optimal. Ensuring patients receive high quality, accessible information regarding their condition and priorities of care is challenging during this time. This project aimed to develop and evaluate the utility and usability of a new, co-designed web resource. Mixed methods co-design principles were employed over a 3-phased study. In Phase 1, content analysis was undertaken of qualitative interviews with 8 patients, 2 carers and 9 multidisciplinary healthcare professionals regarding their experiences during and after AECOPD. Results informed a two-round web-based Delphi survey involving 20 international expert clinician-researchers in Phase 2. Consensus items informed web resource construction in Phase 3, with alpha testing evaluations undertaken by Phase 1 stakeholder participants and beta testing evaluations undertaken of the revised web resource following real-world tablet-device pilot implementation by inpatients during and after AECOPD and their treating therapists. The final resource was presented via webinar to community clinicians and final feedback was captured via online survey. 53/58 items reached consensus during the Delphi survey. Tablet-administered education was feasible. Surveys were completed by 25 inpatients. 80% found the resource useful, 72% reported an increased willingness to participate in pulmonary rehabilitation and 28% indicated they gained new insights or motivation for behavioural change to improve disease management. Follow-up patient evaluations were limited by loss to follow-up (14/25; 56%). Therapists indicated high levels of satisfaction with the resource and perceived improved clinical work efficiencies. Of 21 community clinicians, the median response was ‘strongly agree’ that they ‘would recommend this web resource to patients and/or their carers’. Lung Flare Care is a new web-based educational resource that can aid the delivery of high quality education for best care for people with AECOPD. Further research is needed regarding best implementation recommendations and impact of this novel web resource.

## Introduction

Chronic obstructive pulmonary disease (COPD) is a common global condition (estimate of prevalence ranges between 7.8% to 19.7% of total major city populations [1]) that is expected to remain among the top 10 causes of death and disability-adjusted life years for the foreseeable future [2]. It incurs significant healthcare costs (an estimated $40 billion per year has been projected over the next 2 decades in the United States [1]; 56% of the total respiratory disease healthcare budget is allocated specifically to COPD in the European Union [3], with the greatest contributing factor to total COPD healthcare expenditure being acute exacerbations of COPD (AECOPD) [4].

AECOPDs may occur for a variety of reasons [5] and have negative impacts on health and quality of life, disease progression, risk of re-hospitalisation and mortality [6–8]. They are characterised by a prolonged worsening of respiratory symptoms (e.g., cough, sputum, breathlessness) and functional decline that is not guaranteed to recover spontaneously [9,10]. The extent and timing of recovery from AECOPD is also highly variable for many reasons [11], however incomplete recovery from AECOPD is a significant problem as it predicts risk of re-exacerbation, readmission and short-term mortality [12–14].

The precise method(s) to optimize recovery from AECOPD are not clearly defined. For example, pulmonary rehabilitation (PR) is recommended to commence within 2 weeks of discharge after AECOPD and is highly effective at improving readmission risk, exercise capacity and quality of life [15–18]. However, engagement with and completion of PR after AECOPD is very challenging [19,20]. Many factors interplay to create a complex therapeutic landscape during and shortly after AECOPDs. These include patient related barriers (e.g., lack of understanding and/or perceived benefit of treatment recommendations) [21], cognitive dysfunction during AECOPD [22], psychosocial barriers (e.g., anxiety, distress) [23], suboptimal quality and timing of practitioner care during AECOPDs, challenges to deliver patient education tailored to individuals’ needs, preferences and concerns [24,25], as well as healthcare system and transport complexities [26,27]. Initiatives to improve awareness, knowledge and uptake of effective interventions including PR are now one of the highest global priorities [16].

A pragmatic approach to overcome many of these challenges is to make high quality on-demand educational information available to patients and therapists involved in managing AECOPDs to use as needed at the time of their choosing. Digital platforms afford many benefits including ease of access and presentation of information via multiple media formats (e.g., text and visual animations, patient video testimonies), which are important to help overcome known low levels of health literacy in COPD [28]. The modern landscape of digital design suggests that co-design approaches that include the involvement of and feedback from end users throughout key stages of development and prototype testing to ensure solutions align with needs yield better product utility and usability. The co-design approach enables the creation of digital solutions that address real-life needs, complexities and preferences, and are practical and fitting to the user’s lifestyle or workflow [29,30]. Using a co-design approach to develop a digital health solution therefore seemed fitting for COPD as it is a complex and heterogeneous disease involving many stakeholder groups. No specific evidence exists, however, to help inform the design of a custom resource aimed specifically at educating patients about the importance of optimizing recovery after AECOPD. We therefore sought to address this gap via global engagement of expert stakeholders (e.g., patients and carers, healthcare professionals and researchers) to identify the priority issues and methods to incorporate in such a resource following best practice methods of co-design and consensus development.

The primary aim of this study was to construct a high quality, stakeholder informed web resource designed to facilitate optimal recovery from AECOPD. The secondary aim was to assess the perceived usefulness and utility of the web resource in real clinical practice.

## Materials and methods

### Study design

**Fig 1:**
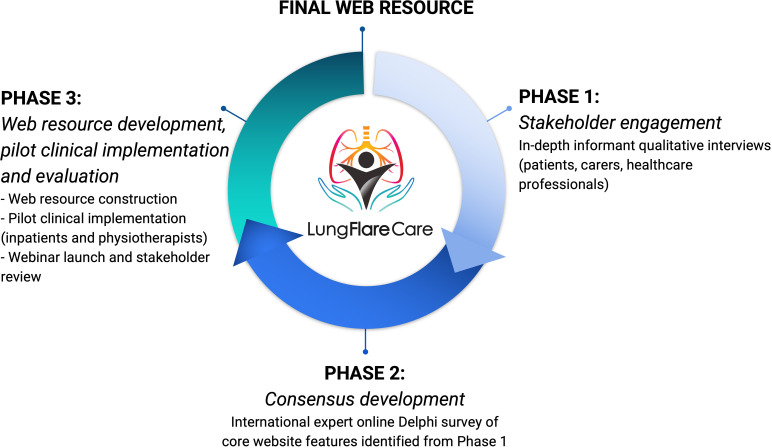
Three phases of the Lung Flare Care study. A mixed methods design.

A mixed methods approach following the principles of experienced-based co-design (EBCD) [31] was adopted and implemented in 3 distinct phases, depicted in Fig 1 and described further. The development and evaluation process of the web resource was scaffolded against recommended practice involving distinct designing, prototype development, and alpha and beta testing phases [32].

This multi-phase study was approved by the Human Research Ethics Committees of Monash University (#24481) and Monash Health (QA/84167/MonH-2022–304668(v2)), and conducted between July 2020 and January 2023. All participants were supplied with an explanatory statement and provided informed verbal consent (or written consent in Phase 2) prior to participation in the study.

### Recruitment and data analysis

#### Phase 1 – Stakeholder engagement.

Phase 1 was a qualitative study involving semi-structured interviews and content analysis. Purposive sampling targeted participation from 3 key informant groups: (1) Patients or carers of patients currently admitted to an acute hospital with AECOPD (identified by treating physiotherapists, and consented to be contacted after discharge for interview by a member of the research team); (2) community dwelling adults with experience of at least one AECOPD or their carers (identified via public advertisements and/or social media); and (3) healthcare professionals with experience managing patients with AECOPD and/or PR (identified via direct contacts of the research team). No exclusion criteria were stipulated provided data collection methods could take place with English language project personnel.

Data were collected via 30-minute telephone interviews conducted by an investigator (AK) experienced in qualitative research. Semi-structured interview scripts were developed by the research team and piloted on a small number of representative users (resulting in minor edits to clarify expression and question sequence). Open-ended questions were used to draw forthright responses and permit flexibility to respond with follow-up questions to achieve information-rich data relevant to the study’s aims. A copy of the interview scripts is in S1 File. Audio-recorded qualitative data were manually entered and coded in Microsoft Excel and analysed via content analysis [33]. Inductive open coding and subsequent grouping via second level coding was performed (by AK) and data saturation point was achieved. Preliminary emergent themes related to common and priority needs, as well as preferences for information delivery during AECOPD recovery were jointly reviewed and refined (AK and CO), with exemplar quotations located for each theme. Final themes informed a list of issues adopted into Phase 2.

#### Phase 2 – Consensus development.

Phase 2 was an online Delphi study that sought to derive international expert consensus regarding the issues and methods to deliver information for the new web resource. Thirty-two international experts were identified based on having contributed at least two publications in the field of AECOPD and/or PR in the prior two years or known profile of advocacy in the field. Each was emailed directly and invited to participate in the Delphi, with additional information provided about how to voluntarily participate in the survey. Two iterative rounds of voting were conducted using Qualtrics Software (version 2020; Provo, UT) with participants rating 51 Delphi items according to a 9-point Likert scale (1 = low; 9 = high) according to perceived importance of topic and perceived effectiveness of delivery media (e.g., imagery, text, layout). A copy of the initial survey is provided in S2 File. Consensus criteria for inclusion was denoted by an item score of ≥7 among ≥80% of responses and an interquartile range of +/- 1. This information was not disclosed to participants. Consensus items informed the construction of the draft web resource for use in Phase 3.

#### Phase 3 – Web resource development, pilot clinical implementation and evaluation.

Phase 3 comprised 3 sub-phases. Phase 3a included drafting, editing and sourcing feedback from multiple stakeholder perspectives (‘alpha’ testing). This involved re-interviewing a representative cohort of informants from Phase 1 (by AK) using a standardized interview script over the phone (S3 File). Content analysis (by AK) was applied to interview answers and open-ended feedback received via email from healthcare professionals, to identify web resource aspects that had met user needs and preferences, as well as areas for revision for the next phase of evaluation.

Phase 3b involved ‘beta’ testing the prototype web resource by patients and healthcare professionals in clinical practice during the time of a hospitalised AECOPD to determine how well it addressed its intended aims. This was undertaken as a Quality Assurance Project at a multi-site metropolitan public health service in Melbourne, Australia. A convenience sample of patients admitted with AECOPD were sequentially recruited by Physiotherapist investigators (CG, GR, LP, CL, AC, RS, MC, JD) guided by standardized therapist instructions using a project script. Patients had a minimum of conversational level of English and were not screened for digital literacy when invited to access and review the web resource via a tablet-device loaned to them for at least 24 hours. A QR code was also provided on business cards to allow users to access the resource via mobile phone (if preferred, and for access following discharge). Feedback was sourced by physiotherapist investigators via paper-based surveys (involving multiple choice and short answer questions, detailed in S4 File) from treating therapists and patients on discharge, as well as patient telephone follow-up 2–4 weeks later (by AK).

Phase 3c involved the launch of the final version of the web resource via a public webinar presentation led by the project team in conjunction with a guest expert presenter from Canada. Participation at this event was invited via a range of dissemination methods including social media posts, direct emailing of known interested colleagues, ‘snowballing’ emails disseminated by webinar presenters and direct advertisements via Lung Foundation Australia (partner organisation for the project). Community perceptions were captured through voluntary completion of an online survey (S5 File) that was emailed out to webinar participants after the online launch. Participants were asked to rate 5 statements pertaining to web resource usability and perceived usefulness according to a 5-point Likert scale (1 = Strongly Agree to 5 = Strongly Disagree) and provide feedback via free text as desired. Website analytics recorded during the time of the clinical implementation were also reviewed. All data were collated in Microsoft Excel and analysed using Stata Statistical Software (Release 17.0; College Station, TX: StataCorp. LLC).

## Results

### Phase 1 – Stakeholder engagement

Nineteen key informants (Table 1) completed interviews.

**Table 1:** Phase 1 participant characteristics.

	Patients and Carers	Healthcare Professionals
Participants (n)	Patient: 8 Carer: 2	9
Sex (female, n)	4	8
Context	AECOPD: 3	Acute healthcare setting: 5
	Stable disease: 7	Subacute/ community setting: 4
Profession (n)	N/A	Medical physician: 2
		Acute physiotherapist: 2
		Rehabilitation physiotherapist: 2
		Acute respiratory nurse: 2
		Community respiratory nurse: 1
Years of experience	<1–14	6–18
related to COPD		

**Textbox 1:** Summary of key themes and supporting quotes from Phase 1 patient and carer interviews.

**Key themes:** Limited knowledge was disempowering (category 1, 4) *Quote: ‘(The) hardest moments for carers are the lead up events prior to calling an ambulance… (and when it arrives) that’s when you feel they’re safe, they’ll be taken care of… and when they get discharged from hospital… You have to deal with the flares of their symptoms yourselves and it’s unclear what to do. What are other options aside from calling the ambulance?... Instructions for the patient and family are not sufficient when they leave hospital.’ – KM (carer)* Life impacts were broad (category 1, 4) *Quote: ‘A word to families and carers on what to expect and how to prepare or plan for impacts on your life… prompts to think about other areas the disease affects beyond the patient’s health… (the) difficulty of watching a loved one in suffering or slow deterioration over time… (being informed that) this is what planning you need to do for long term’. – KM (carer)* Reputation matters: desire for reputation and trust (category 1, 3, 4) *Quote: ‘I always take in what the Specialist said to me and what the Physio said. I’d probably take that more to heart… whatever they tell me to do, then I’ll do that...’ – AT (patient with AECOPD)* Information needs were fluid, often unpredictable (category 1, 2, 4) *Quote: ‘(You are) focused on your medical issues at that point in time (during an admission), only now you perceive questions relating to recovery following discharge. (It) would be good to have something to refer back to.’ – JL (patient with AECOPD)* Specific information desired in one place (category 2, 3) *Quote: ‘I struggle because I’m not a computer person… and you type up “lung disease”, well which one? … you might go into several different kinds… If you go into a website and they have those Q&A questions… ‘What is this? How does it affect you’… a multiple-choice (survey) thing in really plain English, then you can get into (the information you seek) that way’ – JL (patient with AECOPD)* Varied preference for website styles but an optimistic/positive tone desired (category 3, 4) *Quote: ‘It’s got to be very much about positive messaging from the start. We need to get away from the old-school of ‘there’s no cure for this disease that kills over three million people a year’… and you read all of these things that are all negative messaging… They might be factual and comments between a doctor and a patient. When we come to a website where we’re trying to promote a better quality of life for people, it’s got to be positive messaging that is the first thing you see…’ – RW (patient with COPD)*

Content analysis of patient interviews resulted in 137 first-level codes that were subsequently organised into 4 categories (below), with key themes and supportive quotes presented in Textbox 1:

1. Education content regarding COPD disease knowledge including treatment, self-management especially prior to and immediately following hospital admission, implications of disease progression and access to support services (56% [77/137] of codes);
2. Preferred methods of education delivery (14% [19/137] of codes);
3. Preferences regarding the appearance of online health information web resources (look and feel) (18% [24/137] of codes), and;
4. Perceived motivational and dissuading strategies for buy-in of education (12% [17/137] of codes).

An example of the coding tree generated from Phase 1 (patient interviews) is provided in S6 Fig.

Content analysis of healthcare professional interviews resulted in 144 first-level codes subsequently grouped into 5 categories:

1. Patient education content relevant to managing the post-exacerbation recovery period (15% [21/144] of codes);
2. The importance of non-pharmacological strategies and disease-benefiting lifestyle habits in chronic lung disease management (37% [53/144] of codes);
3. Facilitators and barriers to patients adopting health care advice into practice (24% [35/144] of codes);
4. Education content regarding disease knowledge such as the pathophysiology of COPD and management principles (5% [8/144] of codes);
5. Opinions about the user-experience and utility of web resources, and preferred education mediums (19% [27/144] of codes).

### Phase 2 – Consensus development

Of 32 invited experts, 25 completed round 1 (10 male, 6 Australia, 4 North America, 1 South America, 14 Europe) and 20 completed round 2. Following the first round, 6 of the 51 items had reached consensus, 9 items were revised based on participant feedback, 7 new items were added, and 1 section title was amended. Consensus criteria was ultimately reached for 53 of 58 items listed in Table 2.

**Table 2:** Delphi items that did and did not reach expert consensus criteria.

Items that reached consensus^a^	Items that did not reach consensus^a^
Section 1: Disease knowledge	
✔ Understanding COPD (e.g., causes, diagnosis, classification) ✔ COPD management and treatment goals (including role of vaccinations, physical activity and nutrition) ✔ Common medications and correct use of inhalers ✔ Role of oxygen therapy ✔ Causes and management of common respiratory symptoms (e.g., breathlessness, cough) ✔ Causes and management of common extrapulmonary symptoms (e.g., fatigue, pain, cognitive changes) ✔ Importance of smoking cessation ✔ Understanding and optimizing mental health and emotional well-being	X COPD related co-morbidities and their management X COPD related co-morbidities and their management X Management and considerations for advanced disease (including the role of palliative care for severe symptoms) X Role of key multidisciplinary healthcare providers X Role of non-invasive ventilation X Role of lung transplantation
Section 2: AECOPDs and their management	
✔ Understanding AECOPDs (e.g., signs and symptoms, definition) ✔ Complications of AECOPDs (e.g., type 2 respiratory failure, anxiety, deconditioning) ✔ Medical management of AECOPDs ✔ Non-pharmacological management of exacerbation symptoms ✔ Optimizing discharge management from hospital ✔ Importance of having a physical activity/ rehabilitation plan ✔ Optimizing mental health related to AECOPDs ✔ Post-exacerbation outcomes: recovery, recurrence and prognosis ✔ Information for carers ✔ Education delivery (e.g., timing, setting, educators, format) ✔ What to do when experiencing an exacerbation (including seeking help)	N/A
Section 3: Rehabilitation after AECOPDs	
✔ Understanding rehabilitation (e.g., its importance and role) ✔ Physical activity during the transition from hospital to community ✔ Detailed insight into PR (e.g., program duration, structure, content) ✔ Evidence for PR after AECOPD ✔ How to access PR (e.g., referral processes, practicalities, transport) ✔ Models of rehabilitation (e.g., centre/ home-based/ telehealth) ✔ Overcoming barriers to undertaking rehabilitation	N/A
Section 4: Supports for AECOPD recovery	
✔ Accessing help for medical care, health and well-being (e.g., GP, non-rehabilitation options) ✔ Individual and family/ carer coping strategies ✔ Accessing support for people living with COPD (e.g., support groups, web forums) ✔ Addressing psychological and social aspects of behavioural change General tips for living with COPD (e.g., coping with tough weather, air pollution, travel planning)	X Safe and effective use of equipment, infection control and maintenance of devices (e.g., gait aids, portable oxygen, nebulisers) X Social activities and interest groups to promote participation (e.g., choir) X Links to support services (e.g., transport, meals, cleaning)
Section 5: Web design elements	
✔ Short real-life videos ✔ Real-life expert testimonials: videos ✔ Real-life patient testimonials: text/ quotes ✔ Real-life patient testimonials: videos ✔ Use of non-authoritative writing tone (a ‘patient’ voice) ✔ Presentation of information: use of a ‘balanced’ perspective (e.g., present both successes and challenges) ✔ Integrating behaviour change principles (e.g., motivational)	X Stock images (staged clinical scenes) X Real-life photographs X Still illustrations (e.g., figures, drawings) X Animations (e.g., cartoons, animated sketches) X Real-life expert testimonials: text/ quotes X Infographics (e.g., data/ graphs) X Printable factsheets X Scientific literature: written summary of findings X Scientific literature: links (only) to sources X Use of authoritative writing tone (an ‘expert’ voice) X Presentation of information: use of an ‘optimistic’ perspective (e.g., focus on success) X Interactive presentation and activities (e.g., expandible/ collapsible texts, quizzes)

✔ Denotes item did reach consensus criteria, and X denotes item did not reach consensus criteria.

Consensus items were used to inform the construction of the web resource titled Lung Flare Care [43] that underwent further alpha and beta rounds of evaluation and revision in Phase 3.

### Phase 3 – Web resource development, pilot clinical implementation and evaluation

For phase 3a, 14 of 21 potential participants (patients and carers, n = 5; healthcare professionals, n = 9) provided feedback through alpha testing the resource (S7 File). A range of devices were used to review the mock web resource. There were 117 affirming feedback comments and 59 constructive comments. Constructive comments warranted minor revisions to webpage layouts, wording to enhance patient-facing language, and functionality. A key revision to the web resource was the addition of information on palliative care which was repeatedly raised by carers and on-the-ground clinicians as lacking.

**Fig 2:**
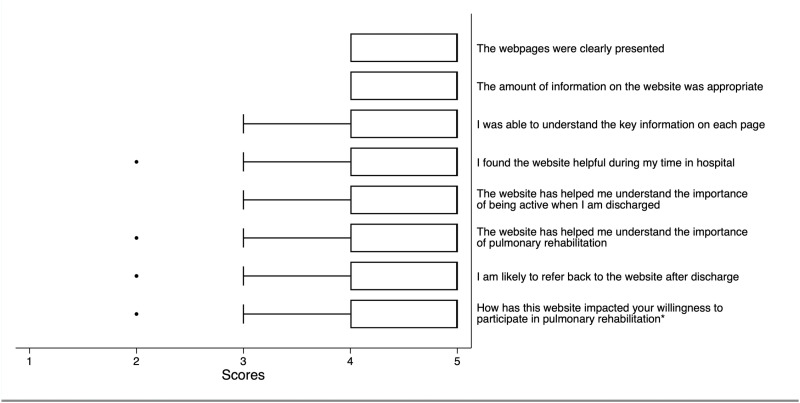
Boxplots of results from web resource evaluation of inpatients. Item responses corresponded to a 5-point Likert scale of agreement (1 = strongly agree; 2 = agree; 3 = neutral; 4 = disagree; 5 = strongly disagree). *Final question response options ranged from 1 (Much less likely to participate) to 5 (A lot more likely to participate).

For phase 3b, 25 inpatients admitted to hospital with AECOPD were recruited and provided inpatient and/or post-discharge feedback (Fig 2). Responses were received from 23/25 (92%) during the inpatient phase but only 14/25 (56%) after discharge due to loss to follow-up. Feedback about the usefulness of each section (1 - ‘About COPD’; 2 - ‘Flare-ups’; 3 - ‘Recovery’; 4 - ‘Support’) was similar, however reduced slightly over the progression from the start to end of the site, possibly related to attrition or the lower observed engagement with those site pages (60%, 56%, 44% and 32%, respectively). High ratings of various aspects of user experience were reported both pre- and post-discharge (e.g., 80% found the resource useful, 28% indicated they gained new insights or motivation for behavioural change to improve disease management) with 18/25 (72%) indicating an increased willingness to participate in PR after viewing the web resource during the inpatient period. Additional data are available in the online dataset [34].

Open-ended feedback responses were categorised into 5 themes; (1) general positive observations, (2) comments indicating intent for behavioural change (e.g., wanting to quit smoking), (3) alluding to gains in disease insights, (4) barriers to information receptivity, and (5) suggestions for the web resource revision. Of note was that more feedback relating to intent for behavioural change was given by the admission group compared to the post-discharge group (22.6% and 11.5% of codes respectively), whereas more suggestions for the web resource revision were offered by the post-discharge group than the admission group (34.6% vs 16.1% respectively).

Website analytics revealed patients spent short periods of time navigating the website during their admissions, predominantly focused on page content related to Understanding COPD, Diagnosing COPD, Disease stages, Medications, and Flare-ups. Less engagement was detected on website sections related to Recovery and Support apart from content for Carer Support and Models of Rehabilitation. Additional details are available in S8 File.

Twenty physiotherapist responses were received from a representative cohort of mixed clinical experiences (junior to senior staff). Ratings of the web resource utility and usability were high from surveyed treating therapists; 95% (19/20) perceived it to be useful for patient education, 80% (16/20) thought it benefited clinical work efficiency and 85% (17/20) felt it had a positive effect on clinical care. Less experienced physiotherapists were more likely not to perceive any effect (positive or negative) on operational efficiency or clinical care.

Twenty webinar participants from Australia and one other from Ireland (n = 21) completed the online feedback survey following the web resource launch. There were high ratings across aspects of the web resource’s perceived usability and usefulness (Table 3).

**Table 3:** Community ratings of the web resource launched.

Statement	Median (IQR)
	score^a^ (n = 21)
The web resource is easy to navigate	2 (1, 2)
The web resource is likely to assist my clinical practice	1 (1, 2)
The web resource contains high quality information	2 (1, 2)
I would recommend this web resource to patients and/or their carers	1 (1, 2)
I would recommend this web resource to other healthcare professionals	1 (1, 2)

^a^Score options correspond to Likert scale statements: (5) Strongly disagree; (4) Somewhat disagree; (3) Neither agree nor disagree; (2) Somewhat agree; (1) Strongly agree.

Seven participants provided feedback via free text which included 3 that offered minor comments to improve website functionality. No feedback warranted significant revisions to the final web resource described in S9 File.

## Discussion

The development of the web resource ‘Lung Flare Care’ [43] represents a step forward in providing patients and therapists with access to high quality, comprehensive, evidence-based information designed specifically to improve recovery following AECOPD. The free web-based resource is multifaceted and multidisciplinary in nature, designed with best practice co-design methodology involving a wide range of stakeholders, and addresses previously identified priority issues in clinical practice including a focus on treatable traits [35], new perspectives on AECOPD management [36], and specific attention on emerging models of PR [37]. We feel the resource is likely to strengthen awareness of evidence-based care for patients but also for healthcare providers. For example, the web resource contains an early recovery plan template (S10 File) designed for clinicians to prescribe early rehabilitation bridging plans whilst awaiting further subacute post-discharge services.

Two essential features of this new resource are (1) the broad range of perspectives sourced from patients, carers, healthcare professionals and academic experts in the field and (2) world-first establishment of expert consensus about how to manage the complexity surrounding management of AECOPDs. The broad perspectives were anticipated as a key driver for undertaking this study and the equality of stakeholder voices was evident in the inclusion of content related to advanced care planning and palliative care in COPD. Although this topic failed to reach the *a priori* consensus threshold during the Delphi survey of academics, it was raised multiple times among patient, carer and healthcare professional stakeholder groups in Phase 1 and Phase 3 feedback and was felt relevant to optimize recovery following AECOPD. Co-design supports this outcome via the equal weight given to different stakeholders and high value placed on the lived experiences of consumers to optimize uptake and engagement of the end product [30]. Additionally, while the development of the web resource commenced with a focus on recovery after AECOPD, strong stakeholder input meant this broadened to encompass a greater range of disease-related educational topics (described in detail in S9 File). Co-design has fast become an essential process when designing applicable and acceptable healthcare solutions [29,31]. While we employed the EBCD model of co-design to achieve this, variability is evident in other studies that have attempted to tackle the complex issues affecting patients with AECOPD. Two UK studies used the EBCD model [35,36] while a Canadian study used the Obesity-related Behavioural Intervention Trials (ORBIT) model [38], each with mixed effectiveness. It is unlikely one approach is ‘right’ or ‘wrong’, acknowledged by the authors themselves [38], however differences in final outcomes based on the underlying approach are plausible on this basis.

## Limitations

Our real-world pilot implementation of the resource highlighted some challenges with resource engagement during the time of AECOPD. Website analytics showed viewing time was low in general (<5 minutes per page) and pages towards the ‘end’ of the resource (requiring more clicks to find) were not commonly accessed. This is perhaps unsurprising considering the vast difficulties patients face during AECOPDs at a time where they experience high levels of cognitive load (including cognitive deficiencies), symptom distress, and high levels of interaction with hospital staff [22]. It also highlights the importance of therapists providing education about how and when to use such a resource to best adapt to patients’ needs at the right time of their choosing. Unfortunately, it was beyond the scope of the implementation project phase to explore the impact of the resource under various models of administration. Further research into patient characteristics including socioeconomic, educational and cultural backgrounds, testing of digital literacy, severity of disease and more in-depth characterisation of past experiences with AECOPD and recovery would also aid the definition of the ideal target user groups who would best benefit from exposure to this web resource at various stages in the patient experience journey.

Whilst the perceived usefulness of the web resource was highly rated by users in this study, feedback was only sought in English language and people with COPD, meaning detailed perspectives were not captured from people from other backgrounds or diseases (however is a current focus of web translation activities and expansion to include asthma and bronchiectasis). Some examples in the web resource were contextualized to an Australian context (e.g., content about living amidst bushfires/natural disasters, video resource examples), however attempts were made to generalize such information wherever possible to optimize relevance in other countries and international health systems (e.g., international PR locator function). Some information was not possible to reconcile (e.g., spirometry cut-offs for diagnosis) due to known differences in the application of such information in different countries. It is also impossible to eliminate the potential for self-serving positive biases when willing participants offer feedback on a resource they were happy to review. Attempts to overcome such issues with neutral question wording and both positive and negative option responses were made to reduce such risks.

The dedicated focus on PR makes this resource an appealing target to attempt to increase uptake and engagement in this critical part of evidence-based health care. The downstream impact on these outcomes was a desired focus of the implementation phase, however it was ultimately deemed unfeasible to evaluate as data collection coincided with many changes to rehabilitation service reorganizations as a result of the SARS-CoV-2 pandemic (e.g., shift towards home-based models, several site-specific programs downsized into a singular ‘hub’ model). The unusual changes meant decisions about engaging patients in such services could not be directly attributed to knowledge or clinical need. In order to address this challenging aspect of clinical care, this resource likely serves a complementary, not substitute, method used in conjunction with other innovations recently being trialled to better engage patients in PR after AECOPDs such as ‘taster sessions’ (i.e., inviting inpatients to undertake short visits to hospital-based PR programs to facilitate better transitions of care) and flexible health service organisation where patients can start and stop PR at various points during their recovery journey across different settings tailored to their needs [39–42]. Therapists may be able to devote more time to such ventures given the perceived time savings reported during the implementation phase of the present study afforded by increased patient agency in their shared decision-making and the opportunity to upskill more junior staff members who may temporarily transition in and out of the respiratory workforce. This is crucial for healthcare providers to note in light of modern pressures to optimize workload efficiency in busy hospital environments.

## Conclusions

Lung Flare Care [43] is a new freely accessible web-based educational resource developed using a multi-stakeholder, interdisciplinary mixed methods co-design methodology. It can assist healthcare professionals to deliver efficient evidence-based education and empower patients to develop agency in shared decision making. Further research is needed to help define target population characteristics and determine its precise best method of implementation to optimize clinically important outcomes such as engagement with PR after AECOPD. Validation of the health and social impact of this resource also remains a topic of future research enquiry. Lung Flare Care [43] can become a vital tool to help improve patient education and potentially outcomes following AECOPD however it will not, nor is intended to, replace the critical role played by healthcare clinicians in the management of these important events.

## Supporting information

S1 File Phase 1 interview scripts. (DOCX)

S2 File Initial Delphi survey. (PDF)

S3 File Phase 3a interview script. (DOCX)

S4 File Phase 3b feedback surveys. (DOCX)

S5 File Phase 3c online survey. (PDF)

S6 Fig Example of Phase 1a coding tree. (PNG)

S7 File Phase 3a summary of feedback from alpha testing. (DOCX)

S8 File Web analytics of inpatient web resource use. (PDF)

S9 File Outline of web resource content. (DOCX)

S10 File Early recovery plan template. (DOCX)
