# Supplementary material for: Lung Flare Care: Development of a web resource to improve recovery after COPD exacerbations: A mixed methods study
Source: PLoS One. 2025 May 22;20(5):e0324468. doi: 10.1371/journal.pone.0324468 (PMC12097615; doi:10.1371/journal.pone.0324468)
Supplement: S1 File — (DOCX) [file pone.0324468.s001.docx]

# S1 File. Phase 1 interview scripts.

## Phase 1a - Patient/Carer Interview Script

Questions for semi-structured, audio-recorded phone interview. Wording to be amended as appropriate for Carers.

**Demographics:**

- No. of years with COPD
- Age, Gender
- Location: State / Region

**About AECOPD experience and Pulmonary Rehabilitation**

1. Can you please tell me about your experiences of AECOPD?
2. What was your experience of returning back to normal physical functioning during the recovery period after discharge?
3. During your AECOPD, how was the topic of physical rehabilitation discussed (if at all)?
4. At the time, what did you think about doing rehabilitation after discharge? Why?
5. Did someone recommend referring you to undertake pulmonary rehabilitation after discharge?
   1. If so, did you accept the offer? Why / why not?
6. What do you wish you would have been informed about regarding PR before you were discharged?

**About information delivery in the recovery period following AECOPD**

1. Do you think this (during AECOPD) was the most suitable time to be informed about physical rehabilitation following discharge?
2. If you were given education about physical rehabilitation after hospitalisation, do you think you would have taken in the information differently? Why?
3. How would you have liked to have received information regarding rehabilitation after AECOPD?
4. What factors, if any, limited your ability to comprehend information regarding rehabilitation provided during hospitalisation?
5. You would receive a considerable amount of information from medical and health professionals. What makes a piece of information stand out to you?
6. What motivates you to act on advice from healthcare professionals?

**About health information gathering preferences**

1. What is your preferred method for finding answers to questions or concerns that you might have about your lung condition?
2. This project aims to develop a new website resource to help improve the delivery of information regarding rehabilitation after AECOPD. Are there any online resources that you have accessed that you find helpful? Please tell us the names and why you found them helpful.
3. Are there online resources that you do not like to use? Why?
4. If you were to access information on a website, what do you think about:
   1. Factsheets / statistics
   2. Animation videos (sketches / cartoons)
   3. Videos (real life demonstrations)
   4. Patient stories

## Phase 1b - Practitioner Interview

Questions for semi-structured, 20-minute audio-recorded phone interview

**Demographics:**

- No. of years working with patients with COPD
- Age, Gender
- Work base: State / Region
- Current role with Patients with COPD:

**Experiences with educating patients with AECOPD**

1. Describe your experience with educating patients following hospitalisation for AECOPD.
2. What do you think are the challenges unique to educating patients recently hospitalised for AECOPD?
3. What strategies have you found successful when attempting to engage with patients regarding rehabilitation after AECOPD?
4. What tips would you give to another clinician for them to have the best chance in educating a patient with recent hospitalisation for AECOPD?
5. How do you try to motivate patients to change their behaviour or comply with advice during/following AECOPD?

**Education content for patients following AECOPD**

1. What do you feel are the most important matters to discuss with patients with AECOPD in regards to their recovery around the time of discharge from hospital? Please elaborate.
2. How important do you feel it is to educate patients about rehabilitation after AECOPD relative to other ‘competing educational topics’ that patients may face during that time (of admission or acute illness)?
3. If it was feasible in your role to educate patients about all aspects of recovery following AECOPD, what other topics would you discuss with them?

**About information delivery in the recovery period following AECOPD**

1. How do you think we should best try to engage with patients regarding rehabilitation in the immediate period following AECOPD?
2. What mediums / platforms do you feel are useful to deliver information / education regarding rehabilitation during this period?
