# Supplementary material for: Lung Flare Care: Development of a web resource to improve recovery after COPD exacerbations: A mixed methods study
Source: PLoS One. 2025 May 22;20(5):e0324468. doi: 10.1371/journal.pone.0324468 (PMC12097615; doi:10.1371/journal.pone.0324468)
Supplement: S2 File — (PDF) [file pone.0324468.s002.pdf]

Introduction

Introduction.
Thank you for agreeing to participate in this online Delphi survey as part of the iRACE project that seeks to **i**mprove **R**ehabilitation **A**fter **C**OPD **E**xacerbations.

Background
iRACE aims to develop a patient-facing educational web-based resource to help deliver high quality, accurate, standardised information regarding rehabilitation in the period directly following acute exacerbations of COPD (AECOPD). You have been invited to participate in this survey based on your expertise and standing in the field. This project has been approved by the Monash University Human Research Ethics Committee (24481), and a copy of the full explanatory statement may be viewed here.

Introduction.
Survey structure
The survey is structured in two parts: 'content' and 'web-design elements'.

Part 1 will ask you to rate the importance of topics to be included in the patient education material for the web-resource. Itemised topics have been informed by preceding qualitative work with consumer stakeholders and are structured according to the following sections:

- General disease knowledge (e.g. COPD diagnosis and management);
- AECOPDs and their management (with a rehabilitation focus);
- Information regarding rehabilitation after AECOPD; and
- Other aspects of supportive care after AECOPD.

Part 2 will ask you to rate the usefulness of different web-design elements to communicate information via the resource. The itemised elements are structured according to the following sections:

- Media types (e.g. imagery, animations, videos, text);
- Incorporation of data and scientific works; and
- Tone, language and communication style.

Voting instructions

You will be asked to rate individual items using a 9-point rating scale, as indicated below:

Low importance / usefulness				Neutral	High importance / usefulness			
1	2	3	4	5	6	7	8	9

Part 1 will involve applying this scale to rate the **importance** of items, while Part 2 will involve using the scale to rate your perceived **usefulness** of items listed. At the end of each section, you will also be given the opportunity to:

- Suggest additional items to be considered in round two of the Delphi;
- Suggest potentially useful resources to inform web content; and
- Offer any general comments / feedback.

Please note that your progression to the next page will indicate your consent to voluntarily participate in this project.

Thank you for your invaluable contribution to this important work.

Dr Christian Osadnik, on behalf of the research team.

Participant details

Introduction.

In order to provide you with personal data feedback between rounds (comparing your voting scores to the sample median) we need to identify your responses. No linked information will be shared beyond the research team. Please type your name below to assist this process.

First name

Last name

Section 1 - Disease knowledge

Section 1. **Disease knowledge**

Please rate the *importance* of the following items to be included in patient education aiming to improve rehabilitation after AECOPD.

Item 1.

	Low importance				Neutral		High importance		
	1	2	3	4	5	6	7	8	9
**Understanding COPD (e.g. causes, diagnosis, classification)**									

Item 2.

	Low importance				Neutral		High importance		
	1	2	3	4	5	6	7	8	9
**COPD management and treatment goals**									

Item 3.

	Low importance				Neutral		High importance		
	1	2	3	4	5	6	7	8	9
**Common medications and correct use of inhalers**									

Item 4.

	Low importance				Neutral		High importance		
	1	2	3	4	5	6	7	8	9

	Low importance				Neutral		High importance		
	1	2	3	4	5	6	7	8	9
**Role of oxygen therapy**									

Item 5.

	Low importance				Neutral		High importance		
	1	2	3	4	5	6	7	8	9
**Causes and management of breathlessness**									

Item 6.

	Low importance				Neutral		High importance		
	1	2	3	4	5	6	7	8	9
**Causes and management of cough and sputum**									

Item 7.

	Low importance				Neutral		High importance		
	1	2	3	4	5	6	7	8	9
**COPD-related comorbidities and their management**									

Item 8.

	Low importance				Neutral		High importance		
	1	2	3	4	5	6	7	8	9
**Importance of smoking cessation**									

Item 9.

	Low importance				Neutral		High importance		
	1	2	3	4	5	6	7	8	9
**Understanding and optimising mental health**									

Item 10.

	Low importance				Neutral		High importance		
	1	2	3	4	5	6	7	8	9
**Role of key multi-disciplinary healthcare providers**									

Item 11.

	Low importance				Neutral		High importance		
	1	2	3	4	5	6	7	8	9
**Management and considerations for advanced disease**									

Section 1.
Please suggest any additional items for this section on 'Disease knowledge' to be considered in the next round of the Delphi.
(Optional)

Section 1. Please suggest any potentially useful resources to inform web-resource content related to this section on 'Disease knowledge'.
(Optional)

Section 1. Please note any other comments or feedback for this section on 'Disease knowledge'.
(Optional)

Section 2 - AECOPDs and their management

Section 2.
AECOPDs and their management
Please rate the *importance* of the following items to be included in patient education aiming to improve rehabilitation after AECOPD.

Item 12.

	Low importance				Neutral		High importance		
	1	2	3	4	5	6	7	8	9
**Understanding AECOPDs (e.g. signs and symptoms, definition)**									

Item 13.

	Low importance				Neutral		High importance		
	1	2	3	4	5	6	7	8	9
**Complications of AECOPDs (e.g. type 2 respiratory failure, anxiety, deconditioning)**									

Item 14.

	Low importance				Neutral		High importance		
	1	2	3	4	5	6	7	8	9

	Low importance				Neutral		High importance		
	1	2	3	4	5	6	7	8	9
**Medical management of AECOPDs**									

Item 15.

	Low importance				Neutral		High importance		
	1	2	3	4	5	6	7	8	9
**Non-pharmacological management of exacerbation symptoms**									

Item 16.

	Low importance				Neutral		High importance		
	1	2	3	4	5	6	7	8	9
**Optimising discharge management from hospital**									

Item 17.

	Low importance				Neutral		High importance		
	1	2	3	4	5	6	7	8	9
**Importance of having a physical activity / rehabilitation plan**									

Item 18.

	Low importance				Neutral		High importance		
	1	2	3	4	5	6	7	8	9
**Optimising mental health related to AECOPDs**									

Item 19.

	Low importance				Neutral		High importance		
	1	2	3	4	5	6	7	8	9
**Post-exacerbation outcomes: recovery, recurrence and prognosis**									

Item 20.

	Low importance				Neutral		High importance		
	1	2	3	4	5	6	7	8	9
**Information for carers**									

Item 21.

	Low importance				Neutral		High importance		
	1	2	3	4	5	6	7	8	9
**Timing and setting of education delivery**									

Section 2. Please suggest any additional items for this section on 'AECOPDs and their management' to be considered in the next round of the Delphi.
(Optional)

Section 2. Please suggest any potentially useful resources to inform web-resource content related to this section on 'AECOPDs and their management'.
(Optional)

Section 2. Please note any other comments or feedback for this section on 'AECOPDs and their management'.
(Optional)

Section 3 - Rehabilitation after AECOPD

Section 3.
Rehabilitation after AECOPD
Please rate the *importance* of the following items to be included in patient education aiming to improve rehabilitation after AECOPD.

Item 22.

	Low importance				Neutral		High importance		
	1	2	3	4	5	6	7	8	9
**Understanding rehabilitation (e.g. its importance and role)**									

Item 23.

	Low importance				Neutral		High importance		
	1	2	3	4	5	6	7	8	9
**Physical activity during the transition from hospital to community**									

Item 24.

	Low importance				Neutral		High importance		
	1	2	3	4	5	6	7	8	9

	Low importance				Neutral		High importance		
	1	2	3	4	5	6	7	8	9
**Detailed insight into pulmonary rehabilitation (e.g. program duration, structure, content)**									

Item 25.

	Low importance				Neutral		High importance		
	1	2	3	4	5	6	7	8	9
**Evidence for pulmonary rehabilitation after AECOPD**									

Item 26.

	Low importance				Neutral		High importance		
	1	2	3	4	5	6	7	8	9
**How to access pulmonary rehabilitation (e.g. referral processes, practicalities, transport)**									

Item 27.

	Low importance				Neutral		High importance		
	1	2	3	4	5	6	7	8	9
**Models of rehabilitation (e.g. centre / home-based / telehealth)**									

Item 28.

	Low importance				Neutral		High importance		
	1	2	3	4	5	6	7	8	9

	Low importance				Neutral		High importance		
	1	2	3	4	5	6	7	8	9
**Overcoming barriers to undertaking rehabilitation**									

Section 3. Please suggest any additional items for this section on 'Rehabilitation after AECOPD' to be considered in the next round of the Delphi.
(Optional)

Section 3. Please suggest any potentially useful resources to inform web-resource content related to this section on 'Rehabilitation after AECOPD'.
(Optional)

Section 3. Please note any other comments or feedback for this section on 'Rehabilitation after AECOPD'.
(Optional)

Section 4 - Other aspects of care following AECOPD

Section 4.
Other aspects of care following AECOPD
Please rate the *importance* of the following items to be included in patient education aiming to improve rehabilitation after AECOPD.

Item 29.

	Low importance				Neutral		High importance		
	1	2	3	4	5	6	7	8	9
**Accessing help for medical care, health and well-being (e.g. GP, non rehabilitation options)**									

Item 30.

	Low importance				Neutral		High importance		
	1	2	3	4	5	6	7	8	9
**Safe & effective use of equipment (e.g. gait aids, portable oxygen)**									

Item 31.

	Low importance				Neutral		High importance		
	1	2	3	4	5	6	7	8	9
**Accessing support for people living with COPD (e.g. support groups, web forums)**									

Item 32.

	Low importance				Neutral		High importance		
	1	2	3	4	5	6	7	8	9
**Links to support services (e.g. meals, cleaning)**									

Item 33.

	Low importance				Neutral		High importance		

	1 Low importance	2	3	4	5 Neutral	6	7 High importance	8	9
	1	2	3	4	5	6	7	8	9
**Addressing psychological aspects of behavioural change**									

Section 4. Please suggest any additional items for this section on 'Other aspects of care following AECOPD' to be considered in the next round of the Delphi.
(Optional)

Section 4. Please suggest any potentially useful resources to inform web-resource content related to this section on 'Other aspects of care following AECOPD'.
(Optional)

Section 4. Please note any other comments or feedback for this section on 'Other aspects of care following AECOPD'.
(Optional)

Section 5 - Web-design elements

Section 5.

Web-design elements
Please rate your perception of the *usefulness* of the following items when communicating information to patients via the web-resource. It might be helpful to

consider the ways in which YOU would personally like all the earlier relevant information to be delivered to YOUR patients if you were unable to do so in person.

Item 34.

	Low usefulness				Neutral			High usefulness	
	1	2	3	4	5	6	7	8	9
**Stock images (staged clinical scenes)**									

Item 35.

	Low usefulness				Neutral			High usefulness	
	1	2	3	4	5	6	7	8	9
**Real-life photographs**									

Item 36.

	Low usefulness				Neutral			High usefulness	
	1	2	3	4	5	6	7	8	9
**Still illustrations (e.g. figures, drawings)**									

Item 37.

	Low usefulness				Neutral			High usefulness	
	1	2	3	4	5	6	7	8	9
**Animations (e.g. cartoons, animated sketches)**									

Item 38.

	Low usefulness				Neutral			High usefulness	
	1	2	3	4	5	6	7	8	9

	Low usefulness				Neutral		High usefulness		
	1	2	3	4	5	6	7	8	9
**Short real-life videos**									

Item 39.

	Low usefulness				Neutral		High usefulness		
	1	2	3	4	5	6	7	8	9
**Real-life EXPERT testimonials: text / quotes**									

Item 40.

	Low usefulness				Neutral		High usefulness		
	1	2	3	4	5	6	7	8	9
**Real-life EXPERT testimonials: videos**									

Item 41.

	Low usefulness				Neutral		High usefulness		
	1	2	3	4	5	6	7	8	9
**Real-life PATIENT testimonials: text / quotes**									

Item 42.

	Low usefulness				Neutral		High usefulness		
	1	2	3	4	5	6	7	8	9
**Real-life PATIENT testimonials: videos**									

Item 43.

	Low usefulness				Neutral		High usefulness		
	1	2	3	4	5	6	7	8	9
**Infographics (e.g. data, graphs)**									

Low usefulness Neutral High usefulness
1 2 3 4 5 6 7 8 9

Item 44.

	Low usefulness				Neutral		High usefulness		
	1	2	3	4	5	6	7	8	9
**Scientific literature: written summary of findings**									

Item 45.

	Low usefulness				Neutral		High usefulness		
	1	2	3	4	5	6	7	8	9
**Scientific literature: links (only) to sources**									

Item 46.

	Low usefulness				Neutral		High usefulness		
	1	2	3	4	5	6	7	8	9
**Printable factsheets**									

Item 47.

	Low usefulness				Neutral		High usefulness		
	1	2	3	4	5	6	7	8	9
**Use of authoritative writing tone (an 'expert' voice)**									

Item 48.

	Low usefulness				Neutral		High usefulness		
	1	2	3	4	5	6	7	8	9

Low usefulness Neutral High usefulness
1 2 3 4 5 6 7 8 9

Use of non-authoritative writing tone (a 'patient' voice)

Item 49.

	Low usefulness				Neutral		High usefulness		
	1	2	3	4	5	6	7	8	9
**Presentation of information: use of an 'optimistic' perspective (e.g. focus on success)**									

Item 50.

	Low usefulness				Neutral		High usefulness		
	1	2	3	4	5	6	7	8	9
**Presentation of information: use of a 'balanced' perspective (e.g. present both successes AND challenges)**									

Item 51.

	Low usefulness				Neutral		High usefulness		
	1	2	3	4	5	6	7	8	9
**Integrating behaviour change principles (e.g. motivational)**									

Section 5. Please suggest any additional items for this section on 'Web-design elements' to be considered in the next round of the Delphi.

(Optional)

Section 5. Please note any other comments or feedback for this section on 'Web-design elements'.

Decision to be acknowledged

Final question.
Some of our consumer interviews revealed a desire for experts to be named in order to lend credibility to the resource. We would therefore like to offer you the **optional** opportunity to have your name and affiliation listed as a contributor to the final web-resource. This will **not be linked** to any data you specifically contributed in this survey.

If you **WOULD LIKE** to be acknowledged on the web-resource, please list your preferred affiliation below (institute / university / health service and country only).

If you leave this question blank or type NO in the box below, we will not identify you as a contributor.

Powered by Qualtrics
