# Supplementary material for: Lung Flare Care: Development of a web resource to improve recovery after COPD exacerbations: A mixed methods study
Source: PLoS One. 2025 May 22;20(5):e0324468. doi: 10.1371/journal.pone.0324468 (PMC12097615; doi:10.1371/journal.pone.0324468)
Supplement: S3 File — (DOCX) [file pone.0324468.s003.docx]

# S3 File. Phase 3a interview script

Date: Name:

Gender: Age:

Location: Role (Patient / Carer / Practitioner):

Device(s) used:

**Interview Questions:**

1. Overall feedback on the website?
2. Look and feel
3. What did you think about the OVERALL APPEARANCE of the website?
4. What did you think about the size and contrast of the text font?
5. How easy to scan for information you’re looking for (able to skim through for key parts?)
6. Navigation
7. Did the way information was organised throughout the website make sense?
8. How easy was it to navigate around the website (going to and from sections)?
9. Were there any pages that stood out to you? Perhaps you liked the look of a page, or did not like the presentation?
10. Content
11. Any content lacking?
12. Was the information easy to digest?
13. Further comments about content?
14. The website serves as a gateway to existing reputable health information – do you have any comments about the links to other sites or resources throughout the site?
15. How did the videos work for you on the website?
16. Any comments on compatibility between smart / electronic devices?
