# Supplementary material for: Lung Flare Care: Development of a web resource to improve recovery after COPD exacerbations: A mixed methods study
Source: PLoS One. 2025 May 22;20(5):e0324468. doi: 10.1371/journal.pone.0324468 (PMC12097615; doi:10.1371/journal.pone.0324468)
Supplement: S4 File — (DOCX) [file pone.0324468.s004.docx]

# S4 File. Phase 3b feedback surveys.

## Phase 3b – Beta testing inpatient feedback survey

Date: _____________ Age: ______ yrs Gender: ____________ Study ID: ________________

Please tick the following device(s) that were used to browse the Lung Flare Care website (URL: www.lungflarecare.com):

□ Tablet (e.g.iPad) □ Mobile phone □ Laptop □ Other: ________________

Which section(s) did you find most useful? (Tick all that apply) □ About COPD □ Flare-ups □ Recovery □ Support

Please **select one response** to each of the statements below following review of the Lung Flare Care website:

|  |  | Strongly disagree | Disagree | Neutral | Agree | Strongly agree |
| --- | --- | --- | --- | --- | --- | --- |
| Q1 | The webpages were clearly presented |  |  |  |  |  |
| Q2 | The amount of information on the website was appropriate |  |  |  |  |  |
| Q3 | I was able to understand the key information on each page |  |  |  |  |  |
| Q4 | I found the website helpful during my time in hospital |  |  |  |  |  |
| Q5 | The website has helped me **understand** the importance of being active when I am discharged |  |  |  |  |  |
| Q6 | The website has helped me **understand** the importance of Pulmonary Rehabilitation after discharge |  |  |  |  |  |
| Q7 | I am likely to refer back to the website after discharge |  |  |  |  |  |
|  |  | Much less likely to participate | A little less likely to participate | About the same / no difference | A little more likely to participate | A lot more likely to participate |
| Q8 | How has this website impacted your willingness **to participate** in Pulmonary Rehabilitation? |  |  |  |  |  |

Do you have any other comments about the website including its strengths and areas for improvement?

_______________________________________________________________________________________________________________________________________

## Phase 3b – Beta testing inpatient follow-up survey

Date: _______________ Study ID: ________________

Please tick the following device(s) that were used to browse the Lung Flare Care website (URL: www.lungflarecare.com):

□ Tablet (e.g. iPad) □ Mobile phone □ Laptop □ Other: ________________

Which section(s) did you find most useful since leaving hospital? (Tick all that apply)

□ About COPD □ Flare-ups □ Recovery □ Support

Please **select one response** to each of the statements below following review of the Lung Flare Care website (URL: [www.lungflarecare.com](http://www.lungflarecare.com)) after discharge:

|  |  | Strongly disagree | Disagree | Neutral | Agree | Strongly agree |
| --- | --- | --- | --- | --- | --- | --- |
| Q9 | I was able to understand the information well |  |  |  |  |  |
| Q10 | I found the website helpful after being discharged from hospital |  |  |  |  |  |
| Q11 | The website has helped me **understand** the importance of becoming more active now |  |  |  |  |  |
| Q12 | The website has helped me **understand** the importance of participating in Pulmonary Rehabilitation |  |  |  |  |  |
| Q13 | I am likely to refer to this website in the future |  |  |  |  |  |
|  |  | Much less likely to participate | A little less likely to participate | About the same / no difference | A little more likely to participate | A lot more likely to participate |
| Q14 | How has this website impacted your willingness **to participate** in Pulmonary Rehabilitation? |  |  |  |  |  |

Do you have any other comments about the website including its strengths and areas for improvement?

________________________________________________________________________________________________________________________________________

## Phase 3b - Treating Physiotherapist Feedback

Position Grade: _____ Date: _______________

Please **select one response** to each of the statements below relating to Lung Flare Care (www.lungflarecare.com):

|  |  | Very unhelpful | Slightly unhelpful | Neutral | Slightly helpful | Very helpful |
| --- | --- | --- | --- | --- | --- | --- |
| P1 | How helpful do you feel it is to have this educational resource accessible for your patients during AECOPD? |  |  |  |  |  |

|  |  | Negative impact | **←** | Neutral | **→** | Positive impact |
| --- | --- | --- | --- | --- | --- | --- |
| P2 | How do you feel this resource impacts on your clinical efficiency? |  |  |  |  |  |

|  |  | Not helpful | **←** | A little helpful | **→** | Very helpful |
| --- | --- | --- | --- | --- | --- | --- |
| P3 | How helpful do you feel this resource is to stimulate patient engagement (i.e. discussions / participation) with physical activity and/or rehabilitation during admission and/or after discharge? |  |  |  |  |  |

Do you have any other feedback relating to Lung Flare Care?

____________________________________________________________________________________________________________________
