# Supplementary material for: Lung Flare Care: Development of a web resource to improve recovery after COPD exacerbations: A mixed methods study
Source: PLoS One. 2025 May 22;20(5):e0324468. doi: 10.1371/journal.pone.0324468 (PMC12097615; doi:10.1371/journal.pone.0324468)
Supplement: S5 File — (PDF) [file pone.0324468.s005.pdf]

Lungflarecare healthcare professionals feedback

Start of Block: Home

Q1
Thank you for registering to attend our recent webinar and website launch for LungFlareCare. In case you have not yet reviewed this new resource, you can find it at: https://lungflarecare.com. We would really value your feedback to find out more about your initial impressions of this new resource.

If you are a healthcare professional, please take a moment to answer the following few questions. This feedback forms part of a project evaluating the development and evaluation of this new resource. All responses are recorded anonymously and you will not be identifiable in any reports that arise from this work (Monash University Human Research Ethics Committee reference #24481).

End of Block: Home

Start of Block: Block 1

Q2 Please select a response to the following statements:

	Strongly disagree (5)	Somewhat disagree (4)	Neither agree nor disagree (3)	Somewhat agree (2)	Strongly agree (1)
The web resource is easy to navigate (1)	○	○	○	○	○
The web resource contains high quality information (2)	○	○	○	○	○
The web resource is likely to assist my clinical practice (6)	○	○	○	○	○

I would recommend this web resource to my patients and/or their carers (4)	○	○	○	○	○
I would recommend this web resource to other healthcare professionals (7)	○	○	○	○	○

Page Break

End of Block: Block 1

Start of Block: Block 2

Q3 In which country do you practice as a healthcare professional?

▼ Afghanistan (1) ... Zimbabwe (1357)

Q4 If you are aware of a pulmonary rehabilitation locator website in your country that might be appropriate to add to our web resource, please paste the url here:

____

Q5 Finally, please use the space below to tell us anything further about Lungflarecare.com *(optional)*

____

____

____

____

____

End of Block: Block 2
