# Supplementary figures and images for: Lung Flare Care: Development of a web resource to improve recovery after COPD exacerbations: A mixed methods study
Source: PLoS One. 2025 May 22;20(5):e0324468. doi: 10.1371/journal.pone.0324468 (PMC12097615; doi:10.1371/journal.pone.0324468)

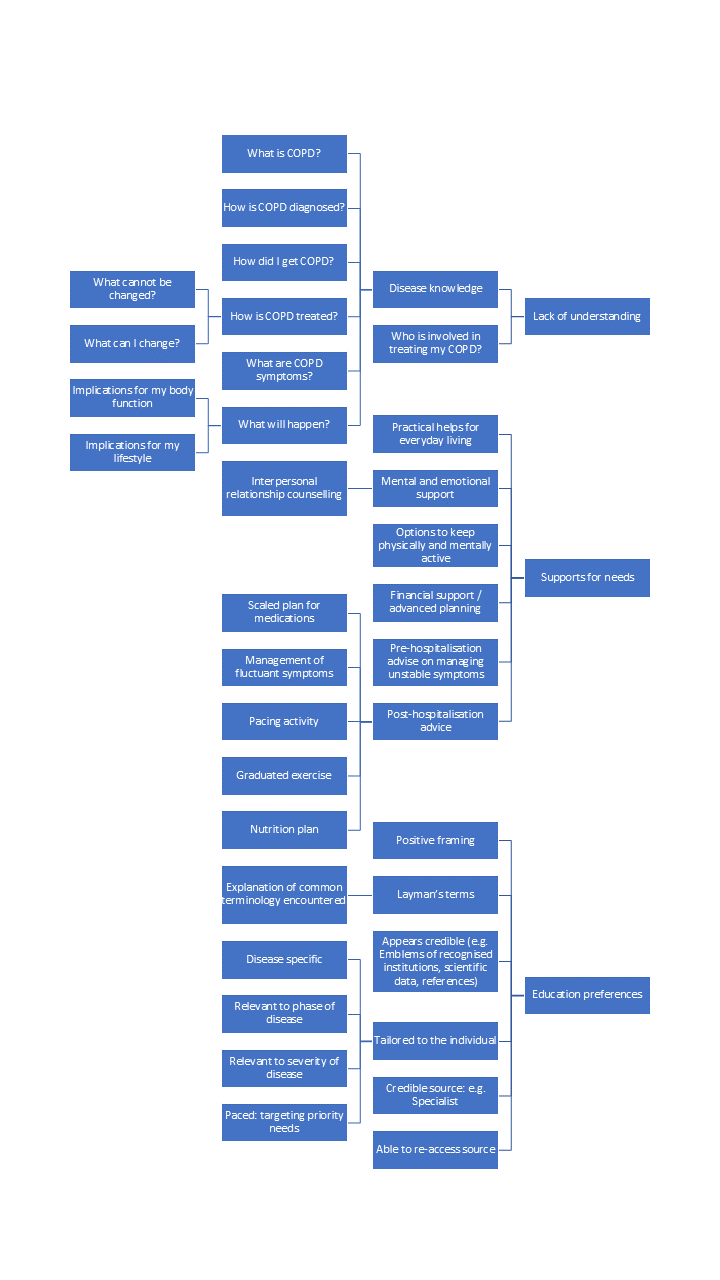

Supplement: S6 Fig — (PNG) [file pone.0324468.s006.PNG]
