# Supplementary material for: Lung Flare Care: Development of a web resource to improve recovery after COPD exacerbations: A mixed methods study
Source: PLoS One. 2025 May 22;20(5):e0324468. doi: 10.1371/journal.pone.0324468 (PMC12097615; doi:10.1371/journal.pone.0324468)
Supplement: S7 File — (DOCX) [file pone.0324468.s007.docx]

# S7 File. Phase 3a - Summary of feedback from alpha testing.

Method of survey and analysis: Phone interview, email correspondence, content analysis

Representative cohort of participants from Phase 1, n=14

- Healthcare professionals: 9 (Includes 4 that gave feedback via email only vs phone interview)
  - Physicians: 1
  - Acute physiotherapists: 2
  - Acute nurse: 2
  - Community nurse: 1
  - Community physiotherapist: 2
  - Community psychologist: 1
- Patients: 4
- Carers: 1

Device(s) used (n): Laptop = 7 iPad = 1 Mobile = 4 Desktop = 6

**Number of feedback comments relating to the following aspects:**

|  | **Aspect** | **Affirmative** | **Constructive** |
| --- | --- | --- | --- |
| 1 | Appearance | 44 | 13 |
| 2 | Content / Language | 33 | 39 |
| 3 | Navigation / Functionality | 14 | 5 |
| 4 | Device compatibility | 4 | 1 |
| 5 | Other | 22 | 1 |
|  | Totals | 117 | 59 |
|  | Total | 176 | |

Description of aspect and example key comments:

1. Appearance – ease of reading (including skimming for key messages), interactive density, visual appeal, look and feel, information architecture per page.

- (Referring to a specific section on the web resource) Too long a paragraph. I’m a skimmer and I might lose interest, just skip the section and move on. – (Carer)

1. Content / Language – appropriateness, accuracy, comprehension, comprehensiveness.

- Content reflects the gap of knowledge in the community and encompasses what patients ask about and what they need to know. – (Patient)
- This is the sort of information when I was first diagnosed I should have seen. A lot of information previously was very negative, about end stage disease, not helpful. This talks about how COPD can be well managed. - (Patient)
- Mention the transition to palliative care. – (Respiratory nurse)
- The use of opioids as part of palliative treatment. – (Physician)

1. Navigation / Functionality – usability and utility of the web resource, organisation or structure of the website.

- Easy to go to the areas you’re interested in very quickly. – (Patient)
- Some people might lose track of where they were at if videos open in the same instead of separate browser. – (Respiratory nurse)

1. Device compatibility – user experience, web resource appearance or functionality pertaining to electronic device(s).

- Thought it’d be harder to view on the mobile phone but it was just as simple, nothing was mis-shaped. – (Respiratory nurse)

1. Other – comments regarding the relevance / potential role / usefulness of this web resource.

- Can result in patients initiating conversations with GP about improving their management. – (Patient)
- I’d be happy to refer clients to look at in the future. – (Respiratory nurse)

Note: Only 3 participants watched videos on the web resource.
