# Supplementary material for: Lung Flare Care: Development of a web resource to improve recovery after COPD exacerbations: A mixed methods study
Source: PLoS One. 2025 May 22;20(5):e0324468. doi: 10.1371/journal.pone.0324468 (PMC12097615; doi:10.1371/journal.pone.0324468)
Supplement: S8 File — (PDF) [file pone.0324468.s008.pdf]

Analytics All accounts > liquidsalt Lung Flare Care Try searching "where did my users come from"

A All Users Add comparison + Custom Jul 1 - Oct 31, 2022

Pages and screens: Page title and screen class

Add filter +

Views by Page title and screen class over time

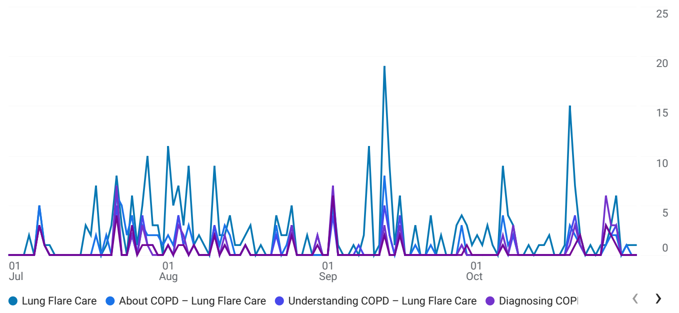

Views by Page title and screen class

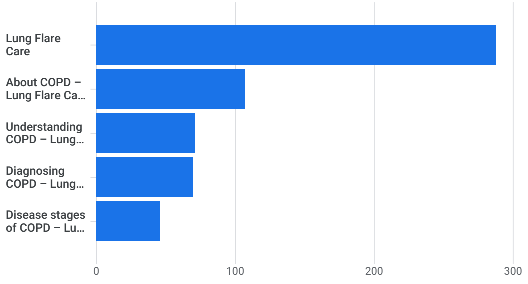

Search... Rows per page: 50 1-34 of 34

	Page title and screen class	Views	Users	Views per user	Average engagement time	Event count All events	Conversions All events	Total revenue
		1,061 100% of total	145 100% of total	7.32 Avg 0%	6m 16s Avg 0%	2,951 100% of total	0.00	$0.00
1	Lung Flare Care	288	132	2.18	0m 58s	861	0.00	$0.00
2	About COPD – Lung Flare Care	107	46	2.33	0m 33s	286	0.00	$0.00
3	Understanding COPD – Lung Flare Care	71	25	2.84	5m 26s	197	0.00	$0.00
4	Diagnosing COPD – Lung Flare Care	70	21	3.33	2m 20s	174	0.00	$0.00
5	Disease stages of COPD – Lung Flare Care	46	18	2.56	3m 13s	122	0.00	$0.00
6	Flare-ups – Lung Flare Care	45	29	1.55	0m 41s	126	0.00	$0.00
7	Healthy habits – Lung Flare Care	42	18	2.33	5m 32s	105	0.00	$0.00
8	Symptoms of COPD – Lung Flare Care	42	21	2.00	2m 23s	111	0.00	$0.00
9	Recovery after a lung flare-up – Lung Flare Care	38	22	1.73	0m 53s	96	0.00	$0.00
10	Pulmonary rehabilitation – Lung Flare Care	34	15	2.27	1m 41s	86	0.00	$0.00
11	Acknowledgements – Lung Flare Care	29	12	2.42	1m 18s	88	0.00	$0.00
12	About flare-ups – Lung Flare Care	23	15	1.53	1m 50s	59	0.00	$0.00
13	Medications – Lung Flare Care	23	13	1.77	4m 25s	61	0.00	$0.00
14	Early management – Lung Flare Care	21	13	1.62	1m 19s	66	0.00	$0.00
15	Treatment and prognosis – Lung Flare Care	20	14	1.43	1m 20s	46	0.00	$0.00
16	Information for carers – Lung Flare Care	17	13	1.31	0m 36s	46	0.00	$0.00
17	Preparing for discharge – Lung Flare Care	17	11	1.55	1m 54s	52	0.00	$0.00
18	Communication – Lung Flare Care	15	12	1.25	0m 59s	37	0.00	$0.00
19	Early Recovery – Lung Flare Care	14	11	1.27	1m 18s	35	0.00	$0.00
20	Lifestyle changes – Lung Flare Care	14	11	1.27	1m 42s	44	0.00	$0.00
21	General information – Lung Flare Care	13	9	1.44	0m 24s	38	0.00	$0.00
22	Rehabilitation models – Lung Flare Care	11	7	1.57	2m 11s	37	0.00	$0.00
23	Accessing pulmonary rehabilitation – Lung Flare Care	10	5	2.00	0m 46s	31	0.00	$0.00
24	Contact us – Lung Flare Care	8	6	1.33	0m 21s	23	0.00	$0.00
25	Peer support – Lung Flare Care	8	7	1.14	1m 29s	23	0.00	$0.00
26	Accessing healthcare – Lung Flare Care	6	6	1.00	0m 33s	19	0.00	$0.00
27	Barriers to rehabilitation – Lung Flare Care	6	5	1.20	0m 35s	17	0.00	$0.00
28	Palliative Care – Lung Flare Care	6	6	1.00	0m 23s	16	0.00	$0.00
29	Carer support – Lung Flare Care	5	5	1.00	5m 51s	15	0.00	$0.00
30	Other considerations – Lung Flare Care	5	4	1.25	2m 55s	17	0.00	$0.00
31	Feedback – Lung Flare Care	3	3	1.00	0m 03s	7	0.00	$0.00
32	References and supportive materials – Lung Flare Care	3	2	1.50	0m 10s	6	0.00	$0.00
33	Search Results for "" – Lung Flare Care	1	1	1.00	0m 20s	2	0.00	$0.00
34	Cuidados com Flare Pulmonar	0	2	0.00	0m 24s	2	0.00	$0.00

© 2022 Google | Analytics home | Terms of Service | Privacy Policy | Send feedback
