# Supplementary material for: Lung Flare Care: Development of a web resource to improve recovery after COPD exacerbations: A mixed methods study
Source: PLoS One. 2025 May 22;20(5):e0324468. doi: 10.1371/journal.pone.0324468 (PMC12097615; doi:10.1371/journal.pone.0324468)
Supplement: S9 File — (DOCX) [file pone.0324468.s009.docx]

# S9 File. Outline of web resource content

Lung Flare Care (URL: <https://lungflarecare.com>) is organised into four main sections aligned with key stages of the patient experience:

1. **About**: contains general information about the disease.
2. **Flare-ups**: contains information about lung flare-ups and their management.
3. **Recovery**: contains information tailored to ensuring individuals understand important issues to optimise recover from their respiratory exacerbation.
4. **Support**: contains content on various topics spanning a range of issues pertinent to attaining good lifestyle habits and chronic disease management

Each section contains multiple sub-section topics and individual sections are hyperlinked with forwards/backwards arrows and floating menus to aid user navigation.

A comprehensive site map of the web resource (including future sections currently under development) is shown in the figure below:


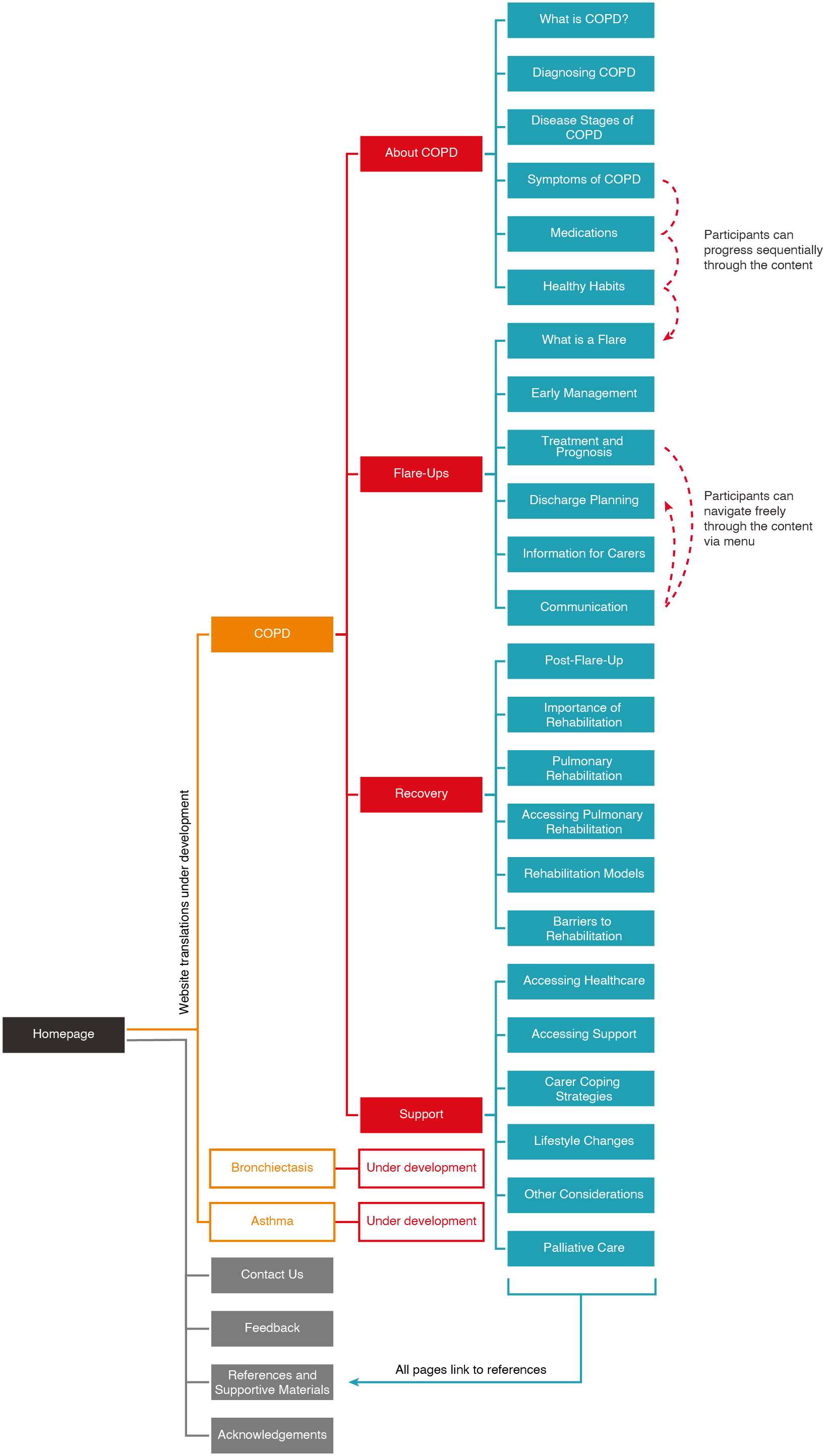


Figure S10a: LungFlareCare site map schema.

Select screenshot examples of the web resource are shown below:


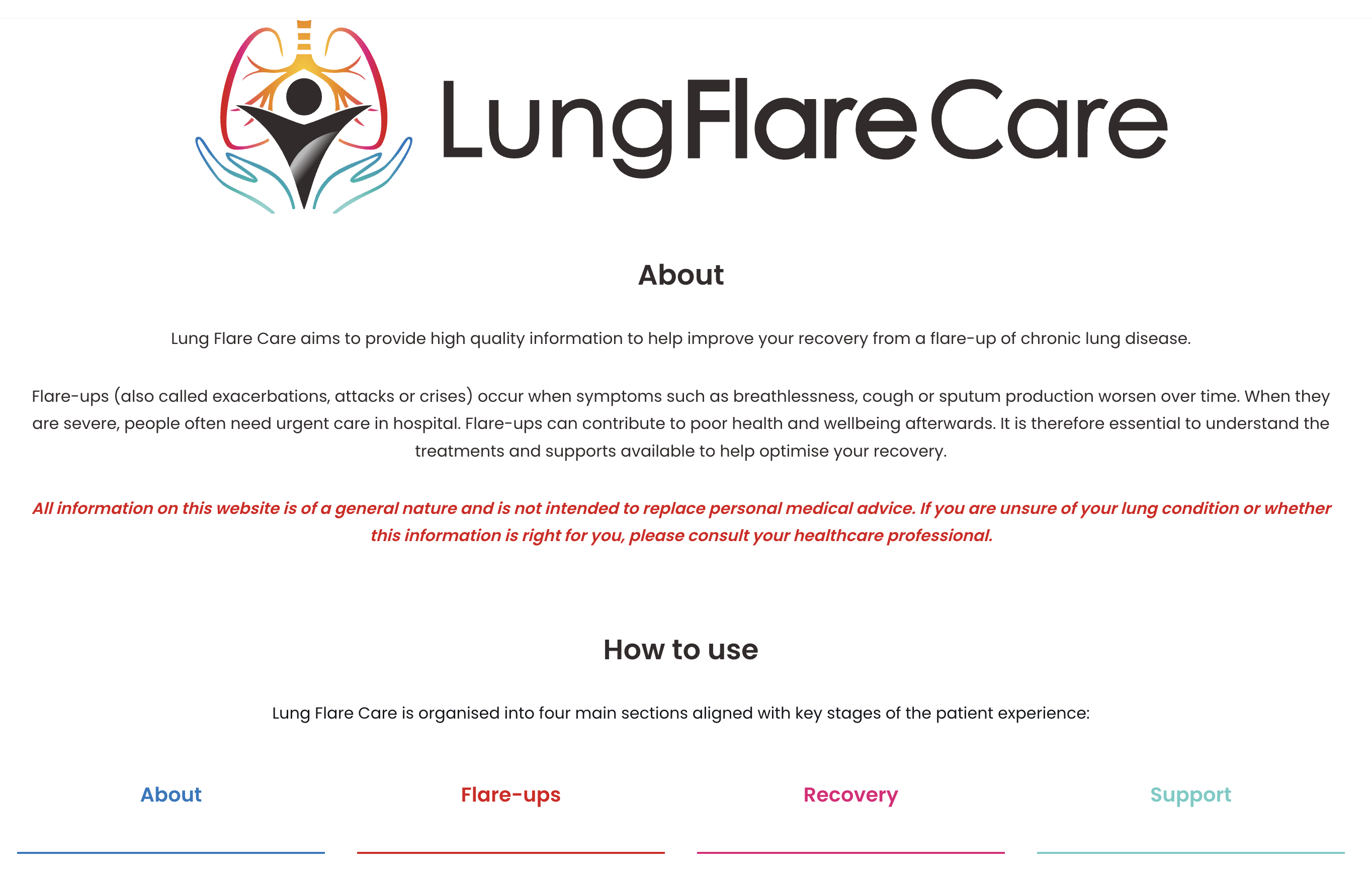


Figure S10b: Home page description of web resource.


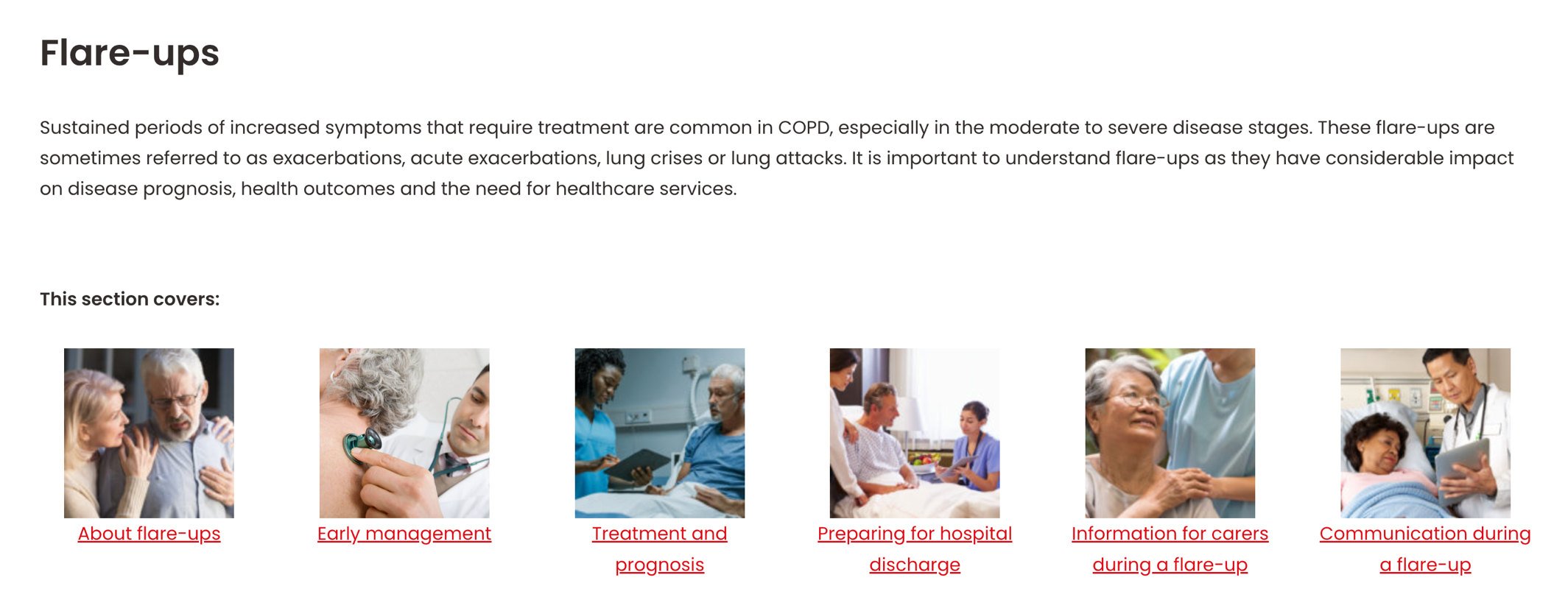


Figure S10c: ‘Flare-ups’ section landing page showing hyperlinked sub-sections.
