# Supplementary material for: Lung Flare Care: Development of a web resource to improve recovery after COPD exacerbations: A mixed methods study
Source: PLoS One. 2025 May 22;20(5):e0324468. doi: 10.1371/journal.pone.0324468 (PMC12097615; doi:10.1371/journal.pone.0324468)
Supplement: S10 File — (DOCX) [file pone.0324468.s010.docx]

#
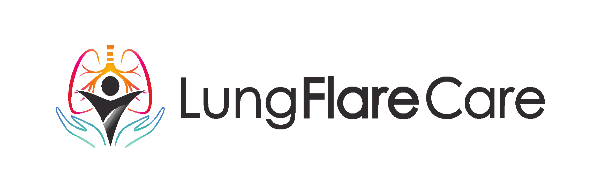
S10 File. Early recovery plan template.

**Early Physical Reactivation Plan**

**My goals**:

In one week, I aim to:

_____________________________________________

In order to achieve this, I will:

- Leave the house ____ time(s) a day, _____ times a week.
- Exercise on the following days of the week (circle):

Mon / Tue / Wed / Thu / Fri / Sat / Sun

My preferred physical activities are:

_____________________________________________

Things I need when undertaking physical activity and/or exercise:

1. Medications (e.g. reliever inhaler)

________________________________________

1. Personal items (e.g. drink / handheld fan / walker / phone)

________________________________________

1. Exercise items (e.g. 2 cans of beans, light dumbbells, step)

________________________________________

**My exercise plan:**

*Walking*:

- Time / distance per walk: _____________________

- Number of walks / day: ______________________

*Strengthening*:


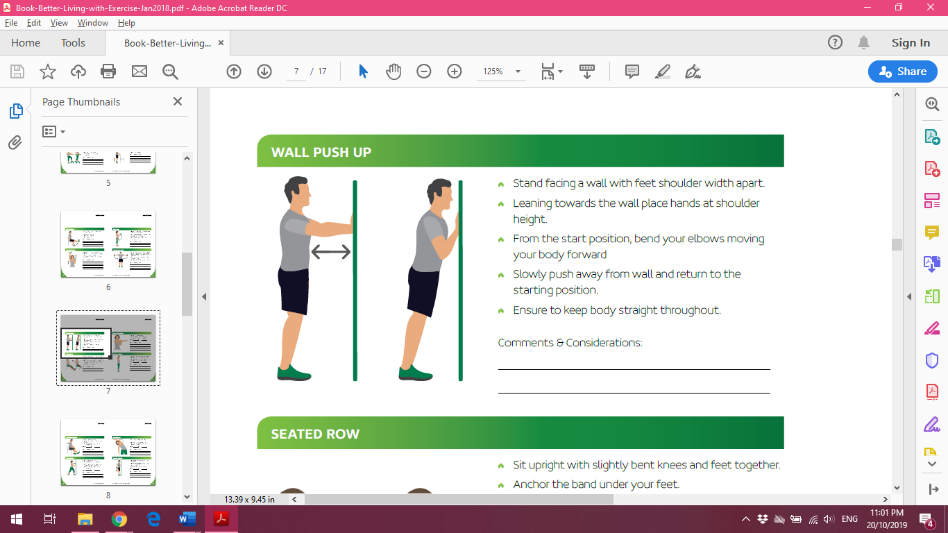

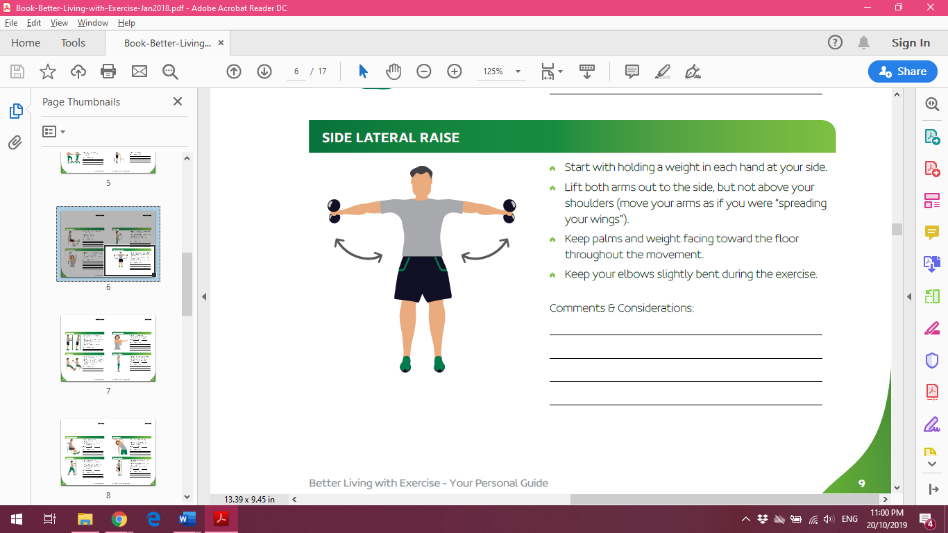

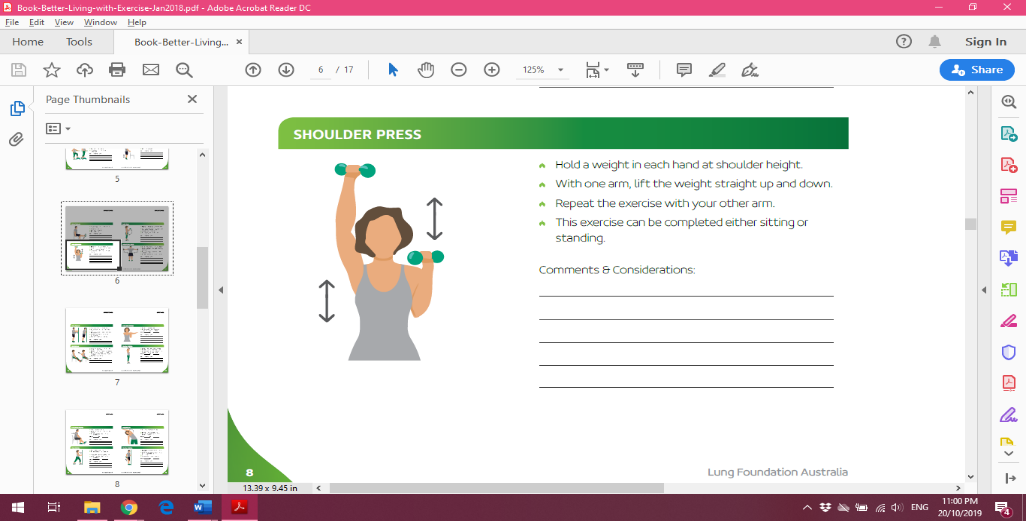
Wall Push-ups Shoulder Press Side Raises


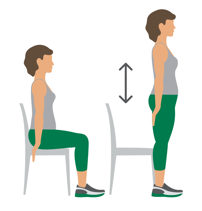

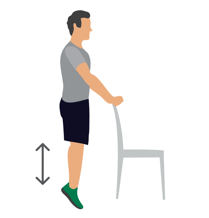


Sit-to-stand Heel raises

- Number of repetitions per session: ________________
- Number of sets per day: _______________________

Who I can talk to about rehabilitation: ________________
